# Novel mutation identified in Leber congenital amaurosis - a case report

**DOI:** 10.1186/s12886-020-01577-9

**Published:** 2020-07-31

**Authors:** Shigeru Sato, Takeshi Morimoto, Sayaka Tanaka, Kikuko Hotta, Takashi Fujikado, Motokazu Tsujikawa, Kohji Nishida

**Affiliations:** 1 Department of Ophthalmology, Osaka University Graduate School of Medicine, 2-2 Yamadaoka, Suita, Osaka, 565-0871 Japan; 2 Laboratory of Regenerative Medicine and Development, Osaka University Graduate School of Medicine, Osaka, Japan; 3 Department of Advanced Visual Neuroscience, Osaka University Graduate School of Medicine, Osaka, Japan; 4 Laboratory of Pathophysiology and Pharmacotherapeutics, Faculty of Pharmacy, Osaka Ohtani University, Osaka, Japan; 5 Special Research Promotion Group, Osaka University Graduate School of Frontier Biosciences, Osaka, Japan; 6 Integrated Frontier Research for Medical Science Division, Institute for Open and Trans-disciplinary Research Initiatives, Osaka University, Osaka, Japan

**Keywords:** RPGR-interacting protein 1, Leber congenital amaurosis 6, Cone-rod dystrophy 13, Inherited retinal degeneration, Case report

## Abstract

**Background:**

Leber congenital amaurosis (LCA) is the earliest onset and the most severe form of all inherited retinal degenerative disorders, characterized by blindness, or severe visual impairment from birth, and typically exhibits clinical and genetic heterogeneity. Recently, 14 causative genes of LCA were reported. We performed whole-exome sequencing (WES) for Japanese siblings, and identified a novel homozygous nonsense mutation in the RPGR-interacting protein 1 (*RPGRIP1*) gene. We also report their follow-up data over 27 years.

**Case presentation:**

Patient 1 is a 37-year-old male. In 1992, his eye position indicated orthophoria, however, horizontal nystagmus was evident, and he complained of photophobia. His best corrected decimal visual acuity (BCVA) was 0.2 (S + 6.5/C-3.5/170°) OD and 0.1 (S + 6.0/C-2.5/10°) OS. Fundus examination revealed bisymmetrical inferior focal retinal pigment epithelium (RPE) mottling. Bright-flash electroretinogram (ERG) revealed a subnormal pattern, while 30 Hz flicker ERG was non-recordable in both eyes. At his final visit in 2019, his BCVA was 0.09 (S + 3.5/C-3.5/180°) OD and 0.09 (S + 3.0/C-4.0/10°) OS. Patient 2, a 34-year-old female, is the sibling of patient 1. In 1992, her BCVA was 0.05 (S + 6.0) OD and 0.06 (S + 5.0) OS. She was in a chin-up position during visual acuity testing. Horizontal nystagmus was evident, and she also complained of photophobia. Bright-flash ERG was severely attenuated, and 30 Hz flicker ERG was non-recordable in both eyes. At her final visit in 2019, her BCVA was 0.02 (uncorrectable) OD and 0.03 (uncorrectable) OS. There were no other patients with LCA in their family and their parents were non-consanguineous. WES revealed a homozygous, consecutive, two-nucleotide variation in the *RPGRIP1* gene (NM_020366: exon15:c.G2294A and c.C2295A, p.C765X), resulting in a premature stop codon. We interpreted this variation as a novel pathogenic mutation of *RPGRIP1* that contributes to LCA6 development.

**Conclusions:**

Herein, we report a novel nonsense mutation of *RPGRIP1* in two patients with LCA6 and present their long-term follow-up data. These clinical data linked to genotypes provide important information for the development of new treatments, such as gene therapy, as well as for genetic counseling.

## Background

Leber congenital amaurosis (LCA) is the earliest and most severe form of all inherited retinal degeneration (IRD), characterized by blindness or severe visual impairment from birth [1, 2]. It is clinically diagnosed as bilateral congenital blindness, with a diminished or absent electroretinogram (ERG) before the age of 6 m, and shows clinical and genetic heterogeneity [1, 2]. Recently, 14 causative genes of LCA were listed in the RetNet (Retinal Information Network) database [3]. These genes encode proteins with different retinal functions, such as photoreceptor morphogenesis, phototransduction, and photoreceptor ciliary transport [2]. However, it is impossible to infer the genotype by ordinary ophthalmic examination. Recent advances in gene therapy have led to the introduction of gene therapy drug approval for LCA2 following identification of an associated mutation in *RPE65*. Therefore, it is important to improve databases for genetic diagnosis with long-term prognosis for each LCA genotype for new treatments and genetic counseling.

In the current study, whole-exome sequencing (WES) was performed for two Japanese siblings suspected to have LCA, or juvenile-onset cone-rod dystrophy, from clinical findings. We also report their long-term follow-up data over 27 years.

## Case presentation

**Fig. 1:**
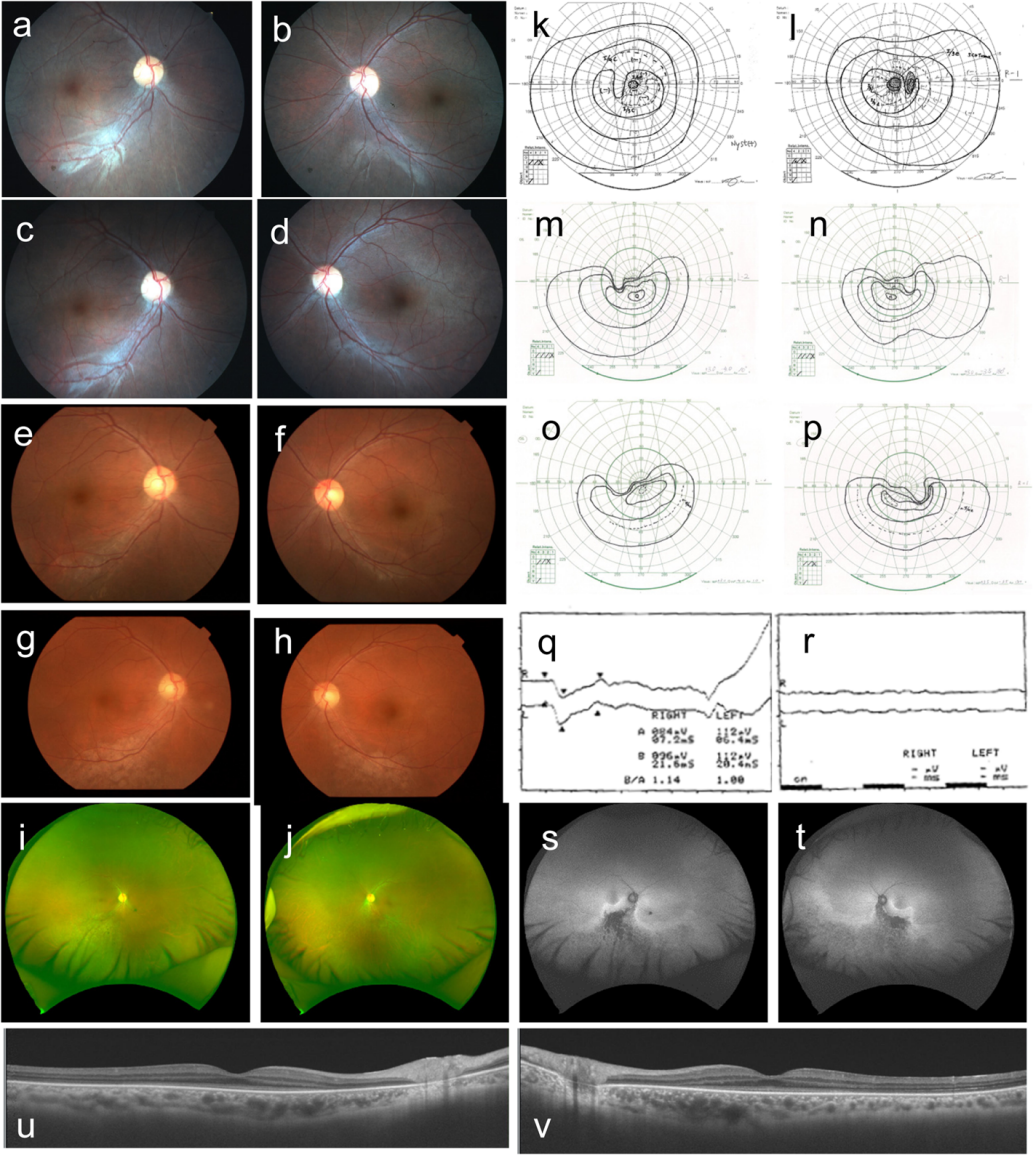
Clinical data for Patient 1. Fundus photos **a** right, **b** left in 1993, **c** right, **d** left in 1996, **e** right, **f** left in 2002, **g** right, **h** left in 2014. Fundus photos of wide field. **i** right, **j** left on 2019. Goldmann perimetry **k** left, **l** right in 2000, **m** left, **n** right in 2014, **o** left, **p** right in 2019. **q** bright-flash electroretinogram (ERG), **r** 30 Hz flicker ERG, upper line was right, lower line was left, respectively. Autofluorescence image of **s** right, **t** left in 2019. Optical coherence tomography (OCT) image of **u** right, **v** left in 2018

Patient 1 is a 37-year-old male who first visited our clinic at the age of 8 years in 1989. His clinical records up to 1992 were not retained, however, a brief summary remained, which indicated that he had nystagmus since birth. Furthermore, he was previously diagnosed with retinitis pigmentosa and hyperopia through clinical examination at the age of 7 at another clinic. Upon his first visit to our clinic, his eyes did not shift in the primary position, and hyperopia and color blindness were detected. Fundus examination was grossly normal. According to the oldest detailed clinical data from 1992, his eye position indicated orthophoria, however, horizontal nystagmus was evident, and he complained of photophobia. His best corrected decimal visual acuity (BCVA) was 0.2 (S + 6.5/C-3.5/170°) OD and 0.1 (S + 6.0/C-2.5/10°) OS. Fundus examination revealed bisymmetrical inferior focal retinal pigment epithelium (RPE) mottling (Fig. 1a, b; photo taken in 1993). Bright-flash ERG showed a subnormal pattern (Fig. 1q; performed in 1998) and 30 Hz flicker ERG was non-recordable in both eyes (Fig. 1r; performed in 1998). At his last visit in 2019, his BCVA was 0.09 (S + 3.5/C-3.5/180°) OD, and 0.09 (S + 3.0/C-4.0/10°) OS. Examining fundus photographs in chronological order (Fig. 1a-j) revealed the gradual progression of bisymmetrical, lower dominant, and focal retinal degeneration. The area of the degenerating retina was more evident with autofluorescence imaging, which showed low fluorescence (Fig. 1s, t). Consistent with this finding, although visual field examinations revealed afferent constriction dominated by upper visual field over time, lower visual field was relatively preserved at 37 years of age (Fig. 1k-p). Optical coherence tomography (OCT) imaging showed that the outer nuclear layer (ONL), ellipsoid zone (EZ), and interdigitation zone (IZ) in the foveal lesion were preserved (Fig. 1u, v: performed in 2018). He had no systemic complications, including mental retardation, hearing loss, or kidney disease noted during the final visit, and he was able to continue his office work.

**Fig. 2:**
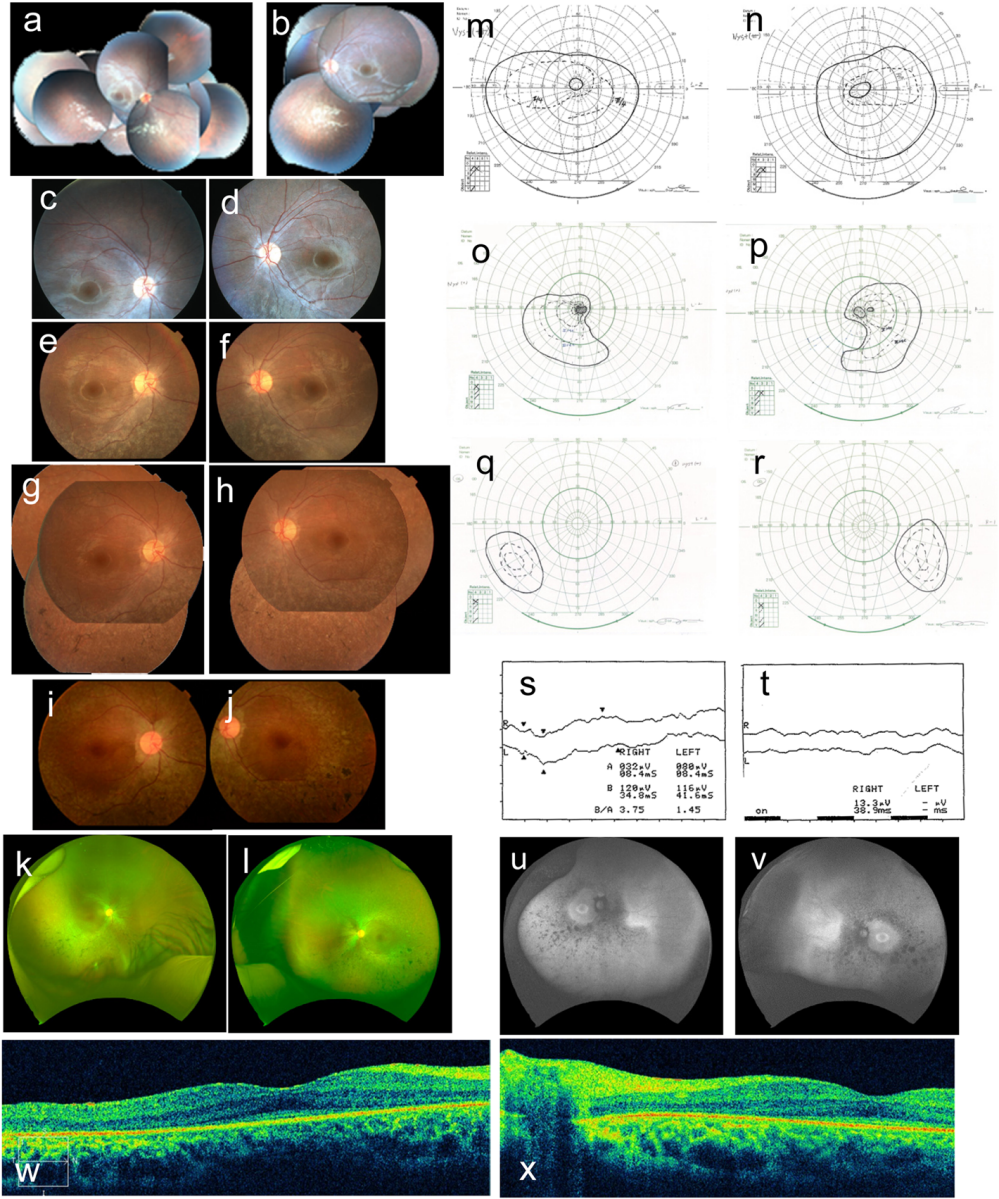
Clinical data for Patient 2. Fundus photos **a** right, **b** left in 1989, **c** right, **d** left in 1993, **e** right, **f** left in 2000, **g** right, **h** left in 2008, **i** right, **j** left in 2017. Fundus photos of wide field **k** right, **l** left in 2018. Goldmann perimetry **m** left, **n** right in 2005, **o** left, **p** right in 2012, **q** left, **r** right in 2019. **s** bright-flash ERG, **t** 30 Hz flicker ERG, upper line was right, lower line was left, respectively. Autofluorescence image of **u** right, **v** left in 2019. OCT image of **w** right, **x** left in 2015

Patient 2, a 34–year-old female, is the younger sibling of patient 1. She first visited our clinic at the age of 5 years in 1989. Her clinical records until 1992 were also not retained, however, a brief summary remained, which indicated that she also had nystagmus since birth. She had already been diagnosed with retinitis pigmentosa and hyperopia through clinical examination at another clinic. She had been wearing prescription glasses since the age 3 years. At her first visit to our clinic, hyperopia was detected. Her fundus photograph at the first visit was retained (Fig. 2a, b). Bisymmetrical inferior dominant retinal pigment epithelium (RPE) mottling without bone spicule pigmentation was evident. According to the oldest detailed clinical data from 1992, her BCVA was 0.05 (S + 6.0) OD and 0.06 (S + 5.0) OS. She was in a chin-up position during visual acuity testing. Horizontal nystagmus was evident, and she complained of photophobia. She failed panel D15 test in 1992. Anomaloscope test was not measurable in 1996. Bright-flash ERG was severely attenuated (Fig. 2s: performed in 1998), and 30 Hz flicker ERG was non-recordable (Fig. 2t: performed in 1998) in both eyes. At her final visit in 2019, her BCVA was 0.02 (uncorrectable) OD, and 0.03 (uncorrectable) OS. Examining the fundus photographs in chronological order (Fig. 2a-l) revealed the gradual progression of retinal degeneration and presence of bone spicule pigmentation. Visual field examination led to suspicion of steady afferent construction in both eyes over time, however, accurate measurement and interpretation was impossible due to nystagmus (Fig. 2m-r). Unlike the older sibling, donut-shaped retinal degeneration was clearly confirmed with autofluorescence imaging of both eyes (Fig. 2u, v) in the younger sibling. OCT imaging showed that ONL, EZ, and IZ in the foveal lesion were preserved (Fig. 2w, x: performed in 2015), however, the preserved lesion was limited compared to her sibling. She also had no systemic complications.

**Fig. 3:**
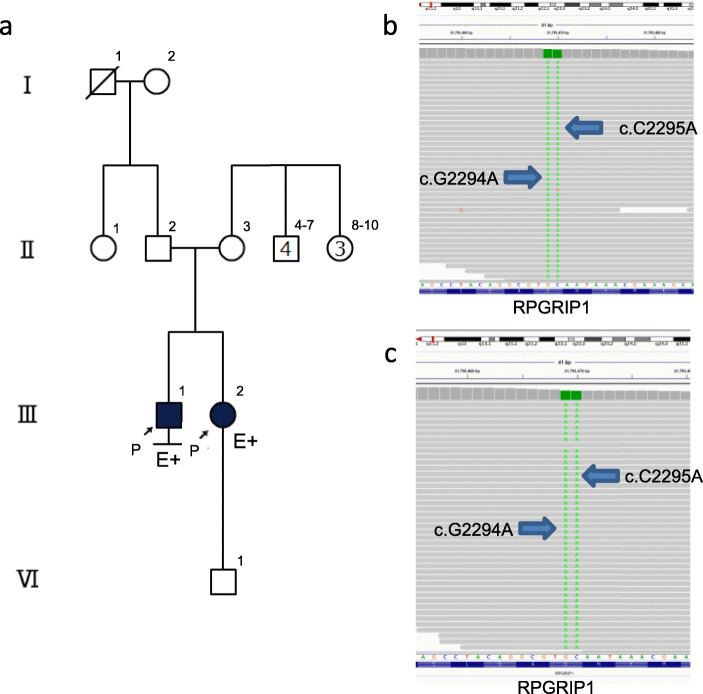
**a** The family tree for the two patients. The parents were non-consanguineous. **b**, **c** The WES data displayed by Integrative Genomics Viewer (IGV). **b** Patient 1, **c** Patient 2

Interviewing the patients revealed that there was no other patient with LCA in their family (Fig. 3a) and that their parents were non-consanguineous. After written informed consent was obtained from the siblings, WES was performed as described previously [4] using Illumina HiSeq 2500 Platform (Illumina, San Diego, CA, USA). At present, we have not yet obtained consent from their parents to participate in this study. Rare variants (minor allele frequency < 0.05) were obtained from the Exome Sequencing Project [5] and 1000 Genomes Project datasets [6], as well as the Human Genetic Variation Database [7]. The candidate variants were identified from the extracted rare variants by referring to the list of inherited retinal degeneration (IRD)-related genes listed in the RetNet (Retinal Information Network) database [3]. The candidate variants were further narrowed down based on whether the siblings had the same variants. Consequently, two candidate variants remained. One was a homozygous, consecutive, two-nucleotide variation in *RPGRIP1* (RPGR-interacting protein 1) (NM_020366: exon15:c.G2294A and c.C2295A, p.C765X, Fig. 3b, c), and the other was a heterozygous, single nucleotide variation in the *NRL* (neural retina leucine zipper) gene (NM_178857:exon4:c.G4006A:p.V1336M). As *NRL*, a bZIP (basic leucine zipper) transcription factor of the Maf (musculoaponeurotic fibrosarcoma) subfamily, is a phosphorylated protein specifically expressed in rod photoreceptors and the pineal gland, and not in cones or other cell types [8, 9], we concluded that the mutation *NRL* could not sufficiently explain the phenotype of our cases. The variation in *RPGRIP1* led to a premature stop codon, as verified by direct sequencing of PCR products (Applied Biosystems 3730 DNA Analyzer; Thermo Fisher Scientific K.K., Tokyo, Japan). The following primer set was used for PCR: c.G2294A and c.C2295A, (forward) 5′-TGATCCTTGCCACACCATCT-3′ and (reverse) 5′-TCATGAGCTGTTTGGCTGAGG-3′. We also investigated the codon conservation using NCBI Orthologs (https://www.ncbi.nlm.nih.gov/gene/3077/ortholog/?scope=32525) and aligned them using the Constraint-based Multiple Alignment Tool (COBALT: https://www.ncbi.nlm.nih.gov/tools/cobalt/cobalt.cgi). Results show that certain mammals, including the chimpanzee, rat, and mouse contain the conserved codon, while dog, bovine, xenopus and zebrafish, did not. Although this variation was not listed in ClinVar [10] or HGMD (The Human Gene Mutation Database) databases [11], it was found to be located upstream of the RPGR (retinitis pigmentosa GTPase regulator)-interacting domain of *RPGRIP1* [12]. Furthermore, pathogenic nonsense mutations were identified downstream of the variation detected in our cases [10, 11]. According to the genome aggregation database (gnomAD v2.1.1: https://gnomad.broadinstitute.org/), the allele frequency of c.G2294A was 0.000004138 (Global), and 0.00 (Asian). Meanwhile, the allele frequency of c.C2295A was unregistered; and the 1000 Genome Browser (https://www.ncbi.nlm.nih.gov/variation/tools/1000genomes/) did not register the allele frequencies of the SNVs. Similarly, the Japanese genetic variation database, HGVD (http://www.hgvd.genome.med.kyoto-u.ac.jp/), and jMorp (https://jmorp.megabank.tohoku.ac.jp/202001/) also did not register them. We, therefore, considered these to be exceptionally rare variants both globally and in the Japanese population. As *RPGRIP1* has been reported as a causative gene for LCA 6 or juvenile-onset cone-rod dystrophy 13 (CORD13) [2], this nonsense variation could explain the phenotype of our cases. We ultimately interpreted this variation as a novel pathogenic mutation of *RPGRIP1* that contributes to the development of LCA6.

## Discussion and conclusions

In this report, we present two Japanese siblings carrying a novel nonsense mutation of *RPGRIP1* with long follow-up clinical data. *RPGRIP1* is reportedly responsible for LCA6 and CORD13 [3]. Although patients with LCA present with visual impairment since birth, and are diagnosed before the age of 6 m, accurate diagnosis during infancy can be difficult. In addition, cases with relatively mild LCA phenotypes caused by *RPGRIP1* mutation have been reported [13]. In our cases, although nystagmus was noted immediately after birth, the diagnosis of retinitis pigmentosa was made at another hospital at the age of 7 and 4 years in patient 1 and 2, respectively. As the age of diagnosis was much later than typical LCA cases, and the clinical data sets at infancy were not available, it was difficult to distinguish between LCA6 and CORD13. However, according to the original report of CORD13, two consanguineous Pakistani families [14] were found to have deterioration in central vision and color blindness from an early age, as well as rapid loss of vision observed between the ages of 14 and 16 years. The mutation in *RPGRIP1* detected in their case was a non-synonymous substitution. Therefore, it was believed that the mutant protein had residual activity, resulting in a mild phenotype. In addition, non-synonymous substitutions are often detected in CORD13, while nonsense mutations are often detected in LCA6 [12, 15]. Taking the above into consideration, our cases were diagnosed as LCA6.

In our cases, nystagmus, low visual acuity, color blindness, and flat 30 Hz flicker ERG responses were observed. In bright-flash ERGs, amplitude significantly reduced, however, was clearly detected at 17 and 14 years of age in the older and younger siblings, respectively. During the 27-year follow-up period, although their visual acuities slightly reduced, they were maintained until their thirties. Retinal degeneration gradually progressed in both patients, however, there was a difference in the speed of progress with the elder sibling exhibiting lower dominant retinal degeneration progression and sectorial retinal degeneration at the final visit. Alternatively, the younger sibling showed lower dominant retinal degeneration progression at her younger age, yet showed typical donut-shaped retinal degeneration with bone spicule pigmentation at the final visit. The cause of this difference in progression should be analyzed in the future. It has been reported that patients with LCA6 often have hyperopia lower than + 7.0 D [15]. Both siblings were hyperopic in childhood, however, bilateral refractive errors in the older sibling diminished with age. Meanwhile, the refractive error of the right eye in the younger sibling also diminished with age (S + 2D/OD, S + 4D/OS: performed in 2009). Hence, in adulthood, high hyperopia might not be a feature of LCA6.

It has also been reported that RPGRIP1 localizes only in the photoreceptor connecting cilium (CC), and is associated with RPGR, known as a causative gene of X-linked retinitis pigmentosa [16, 17]. RPGRIP1 binds to the RCC1 homology domain of RPGR via its C-terminal portion, called the RPGR-interacting domain [12, 18]. In a mouse model lacking *Rgprip1*, RPGR was absent in the CC of photoreceptors, however, the opposite was not observed [16]. Here, we identified a novel nonsense mutation in exon 15 of *RPGRIP1*, which is upstream of the RPGR-interacting domain. Although nonsense-mediated mRNA decay could be expected and if truncated protein produced, it would not interact with RPGR and would not have normal function.

Recently, the effectiveness of gene therapy in mouse and dog LCA models with *RPGRIP1* gene deficiency has been reported [16–21] finding that the retinal laminar architecture was preserved in the central retina, but not in the pericentral or peripheral retina with OCT imaging. From these results they concluded that gene therapy has treatment potential if targeted to the central retina, but not pericentral or peripheral retina, in patients with LCA6 [22]. In our cases, the outer retinal structure in the foveal lesion was more extensively maintained in the third decade, indicating that in some cases, the target site for treatment may be wider and the time window may be longer. Hence, genotyping is a prerequisite for subgrouping genetically defined patients for gene therapy, and the accumulation of clinical data linked to genotypes will enable the development of new treatments. However, at present, most IRDs have no predictable prognosis. It is, therefore, also important to expand the knowledge database of long-term prognosis for each genotype. Such efforts provide essential basic data not only for the development of new treatments, such as gene therapy, but also prognostic information for patients with IRDs to improve genetic counseling.

## Data Availability

All data generated or analyzed during this study are included in this published article.

## Abbreviations

LCA: Leber congenital amaurosis
IRD: Inherited retinal degeneration
ERG: Electroretinogram
WES: Whole-exome sequencing
RPGRIP1: RPGR-interacting protein 1
BCVA: Best corrected decimal visual acuity
CORD13: Juvenile-onset cone-rod dystrophy 13
OCT: Optical coherence tomography
ONL: Outer nuclear layer
EZ: Ellipsoid zone
IZ: Interdigitation zone
RPE: Retinal pigment epithelium
NRL: Neural retina leucine zipper
bZIP: Basic leucine zipper
Maf: Musculoaponeurotic fibrosarcoma
HGMD: The Human Gene Mutation Database
RPGR: Retinitis pigmentosa GTPase regulator
CC: Connecting cilium
